# Supplementary material for: A novel feature-scrambling approach reveals the capacity of convolutional neural networks to learn spatial relations
Source: Neural Netw. Author manuscript; Available in PMC 2024 Nov 26. (PMC7616855; doi:10.1016/j.neunet.2023.08.021)
Supplement: Appendix [file EMS200277-supplement-Appendix.pdf]

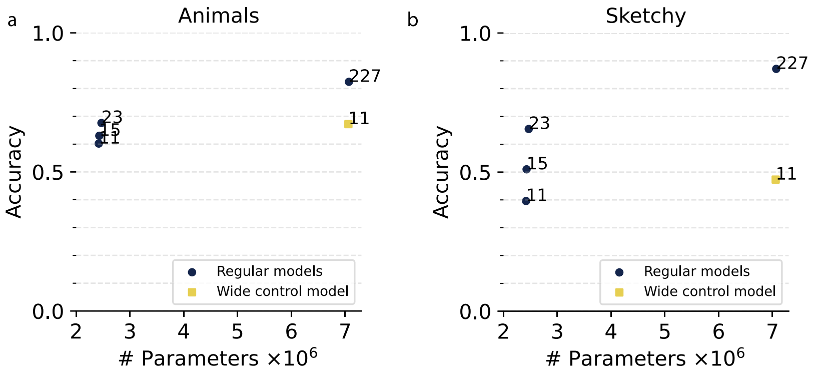

Fig. A.1. A control experiment in which we trained a wide model that has a small ERF (11 pixels), while matching the number of parameters of the model with the largest ERF (227 pixels). The numbers shown in the figure are the ERF of the corresponding models in pixels.

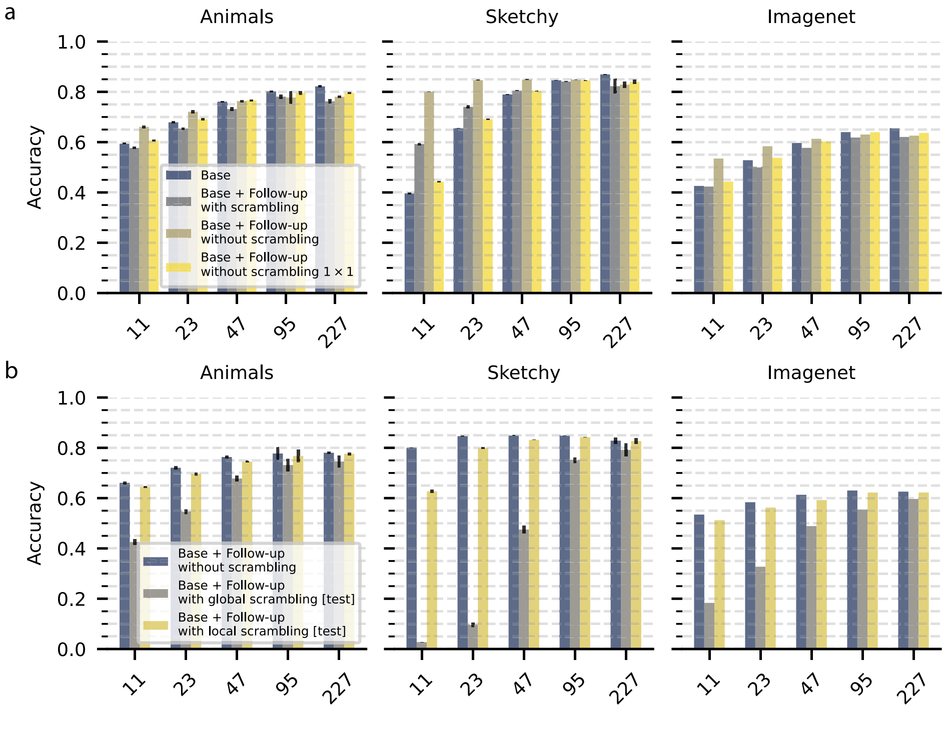

Fig. A.2. (a): Classification accuracy for CNN models of different ERFs under different training conditions of the feature-scrambling approach (Fig. 1d). (b): Classification accuracy of the base models with spatial aggregation without scrambling under different testing conditions (global and local scrambling).

Appendix

Controlling for the number of parameters of the models. We performed a control analysis to verify that the performance differences observed in our study among CNNs of different ERFs can be attributed indeed to their ERFs and not the number of model parameters. We trained a wider model of small ERF (11×11 pixels) but *with* matched the number of parameters to the model with the largest ERF (227×227 pixels). For both the Animals and Sketchy datasets, we observed a slight increase in the classification performance of the models by increasing the number of parameters. However, a small ERF model with a large number of parameters did not reach the performance of the model with the largest ERF, indicating the importance of the ERF to the models' performance. Furthermore, for the Sketchy dataset, the performance of the wider model with ERF $= 11 \times 11$ did not even reach the performance of the regular model with ERF $= 15 \times 15$ pixels. This is in line with our other results showing the reliance of the performance of CNNs on their ERF size,

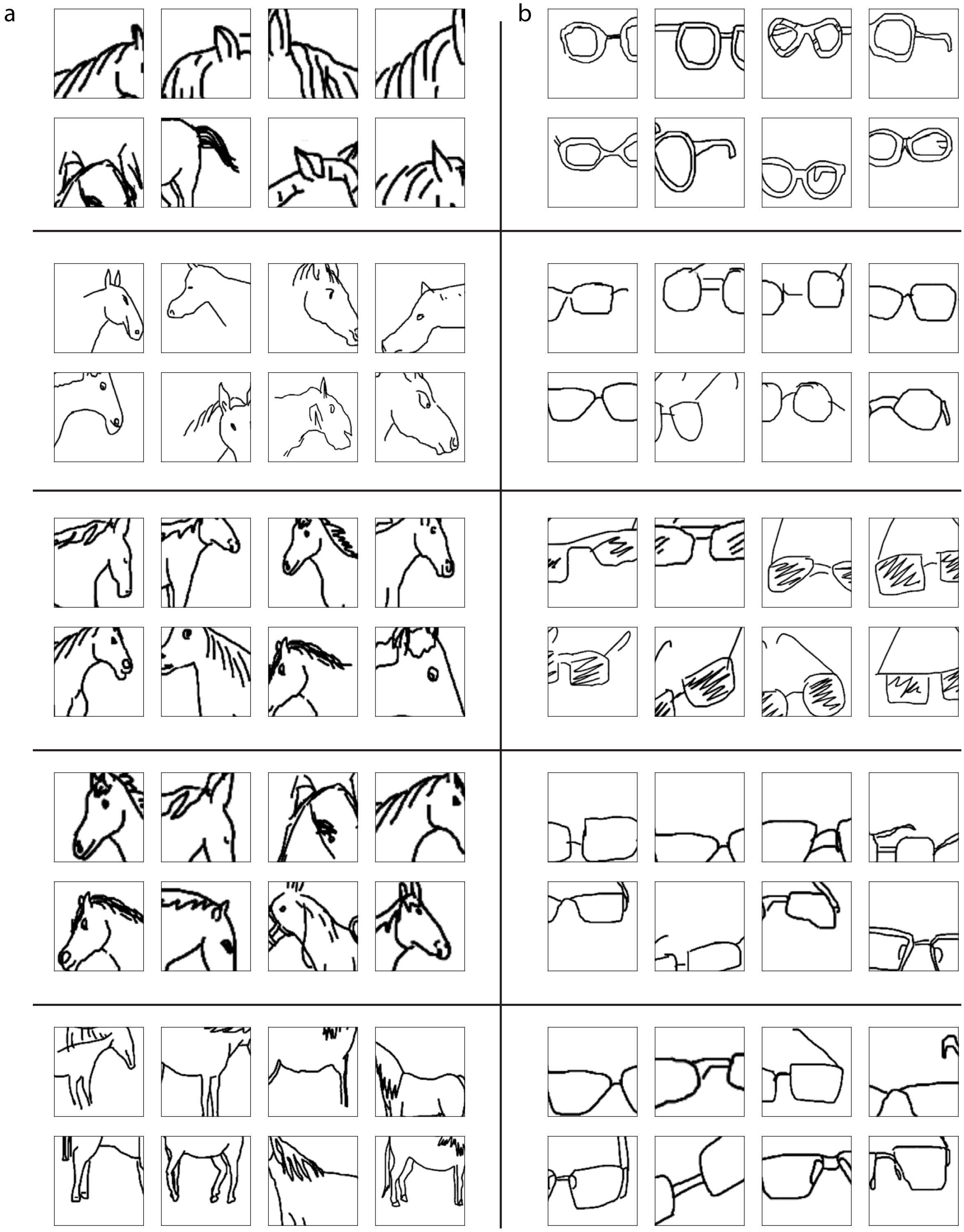

Fig. A.3. Clustering of all the MIRCs of the horse (a) and eyeglasses (b) classes (Sketchy dataset) in the representational space of the model ERF227 Each panel shows the eight closest MIRCs, generated from unique test images, in the representational space to the center of one cluster.

especially for the Sketchy dataset. Note that in the manuscript, we included several additional controls, e.g. scrambling during training, a 1 × 1 follow-up network, and local scrambling, which further show the importance of ERF size.
